
JOURNAL INFORMATION
==============================
NLM Title Abbreviation: ScientificWorldJournal
Journal ID: ScientificWorldJournal
NLM Journal ID: 101131163
Journal ID: TSWJ

The Scientific World Journal

ISSN: 2356-6140
EISSN: 1537-744X
Publisher: Wiley

ARTICLE INFORMATION
==============================
PMCID: PMC6084532
PMID: 30147606
DOI: 10.1100/tsw.2001.230
Article ID: 856298
Article version: 1
Subjects: Short Report

The Oxidative Stress Response Gene P66 and the Tumor Suppressor Gene P53 Induce Mitochondrial Dna Damages

Trinei Mirella
Giorgio Marco
Barozzi Sara
Pelicci Pier Giuseppe *
Department of Experimental Oncology, Istituto Europeo di Oncologia, via Ripamonti 435, 20141, Milan, Italy
*Pier Giuseppe Pelicci: pgpelicci@ieo.it
Publication date: 2001
Electronic publication date: 2001 Oct 12
Volume: 1
First page: 127
Last page: 127
Copyright: Copyright © 2001 Mirella Trinei et al.
Copyright year: 2001
License: This is an open access article distributed under the Creative Commons Attribution License, which permits unrestricted use, distribution, and reproduction in any medium, provided the original work is properly cited.
License URL: https://creativecommons.org/licenses/by/3.0/

==============================
Miami *Nature Biotechnology* Short Reports
*TheScientificWorld* (2001) 1 (S3), 127SR
ISSN 1532-2246; DOI 10.1100/tsw.2001.230

### THE OXIDATIVE STRESS RESPONSE GENE P66$^{SHC}$ AND THE TUMOR SUPPRESSOR GENE P53 INDUCE MITOCHONDRIAL DNA DAMAGES

Mirella Trinei, Marco Giorgio, Sara Barozzi, and Pier Giuseppe Pelicci*

Department of Experimental Oncology, Istituto Europeo di Oncologia, via Ripamonti 435, 20141, Milan, Italy
* pgpelicci@ieo.it

**INTRODUCTION**. The p66$^{Shc}$ gene regulates the oxidative stress response and life span in mammals (1). The p53 gene regulates various cellular responses to environmental stresses, particularly those inducing DNA damage, such as cell cycle arrest, nuclear DNA repair, senescence and apoptosis (2-3). Both p66$^{Shc}$ and p53 increase the intracellular concentrations of reactive oxygen species (ROS), measured by DCFDA staining of wt and -/- PEFs counterparts (our man. in prep). Oxidative stress is considered the principal proximal mechanism of ageing in mammals and lower organisms (4). ROS are also responsible for nuclear DNA mutagenesis and tumor formation.

We report here that p66$^{Shc}$ and p53 expression contribute to the generation of mitochondrial DNA adduct formation mediated by ROS, both in cultured cells and in the mouse tissues.

**METHODS**. To measure mitochondrial DNA adducts we co-amplified a long and a short mtDNA fragments from total cellular DNA by the Extra Long PCR (Perkin-Elmer kit) method. This method is based on the facts that the random DNA alterations induced by ROS reduce the rate of elongation by polymerases and decrease the final yield of PCR products (5-6). A QIAamp kit (Qiagen) purified DNA was employed for the amplification.

**RESULTS**. This technique was applied to determine whether the higher intracellular concentration of ROS in cells expressing p66$^{Shc}$ and p53 (as compared to their corresponding p66Shc-/- and p53-/- control cells) correlate with increased mtDNA damage. We detected increased mtDNA damage by XLPCR in primary wt fibroblasts versus p66Shc-/- and p53-/- fibroblasts. The same difference was also found in mouse adult skin fibroblasts and several different tissue samples.

To investigate whether the increased mtDNA damage was directly associated with the expression of p66$^{Shc}$ and p53, we reintroduced these proteins in p66Shc-/- and p53-/- primary fibroblasts by retroviral or adenoviral infections. XLPCR confirmed the effect of p66$^{Shc}$ and p53 on mtDNA damage.

**DISCUSSION**. Even if the molecular mechanism of ageing and its relation with cellular transformation are not entirely understood, mitochondria are considered crucial sites for both, ageing and transformation. Mitochondria are the major source of free radicals in the cell and mtDNA is the main target of free radicals, whose deleterious effects result in apoptosis, cellular senescence and organism ageing.

The fact that p66$^{Shc}$ and p53 mediate increased ROS production, augmented mtDNA damage, and are both associated to a highly mutagenic intracellular background suggests a possible mechanism of action of these two proteins in the regulation of the senescence program and ageing.

**REFERENCES.**

1. Migliaccio, E., Giorgio, M., Mele, S., Pelicci, G., Reboldi, P., Pandolfi, P.P., Lanfrancone, L., and Pelicci, P.G. (1999) Nature 402, 309-313
2. Giaccia, A.J. and Kastan, M.B. (1998) Genes Dev. 12, 2973-2983
3. Sherr, C.J. and Weber, J.D. (2000) Curr. Opin. Genet. Dev. 10, 94-99
4. Linnane, W., Marzuki, S., Ozawa, T., and Tanaka, M. (1989) Lancet 1, 642
5. Yakes, F.M. and Van Houten, B. (1997) Proc. Natl. Acad. Sci. U S A 94, 514-519
6. Yakes, F.M., Chen, Y., and Van Houten, B. (1996) in Technologies for Detection of DNA Damage and Mutations (Pfeifer, G.P., Ed.), Plenum Press, New York, pp. 169-182
